# Comparative genomics of dairy-associated *Staphylococcus aureus* from selected sub-Saharan African regions reveals milk as reservoir for human-and animal-derived strains and identifies a putative animal-related clade with presumptive novel siderophore

**DOI:** 10.3389/fmicb.2022.923080

**Published:** 2022-08-15

**Authors:** Christoph Jans, Joseph Wambui, Marc J. A. Stevens, Taurai Tasara

**Affiliations:** ^1^Laboratory of Food Biotechnology, Institute of Food, Nutrition and Health, Department of Health Science and Technology, ETH Zurich, Switzerland; ^2^Institute of Food Safety and Hygiene, University of Zurich, Zurich, Switzerland

**Keywords:** *Staphylococcus aureus*, siderophore, iron scavenger, population structure, African dairy products, One Health, neglected tropical disease

## Abstract

*Staphylococcus aureus* infection is considered to be a neglected tropical disease with huge impact on human and animal health alike. Dairy production in sub-Saharan Africa (SSA) relies heavily on various animals such as cows, goats, and camels, depending on the region. *S. aureus* causes mastitis and exhibits high prevalence in raw milk. The population structure including genotypic and phenotypic traits of dairy *S. aureus* in relation to animal and human isolates is, however, unknown for SSA. In this work, 20 *S. aureus* dairy isolates from East and West Africa were selected for comparative genomics and phenotypic analysis. Comparing their population structure revealed a large diversity of different origins suggesting milk to be a reservoir for human and animal strains alike. Furthermore, a novel putative siderophore was detected in multiple strains in a distinct animal-clade with strains of global origin. This putative siderophore shares a high genetic identity with that from *Streptococcus equi* suggesting possible horizontal gene transfer. These findings combined with the virulence genes harbored by these dairy-derived strains such as *pvl*, human evasion factor *scn*, various enterotoxin, leucocidin and antibiotic resistance genes, stresses the need for an integrative One Health approach to tackle the problem of *S. aureus* infections in animals and humans in sub-Saharan Africa.

## Introduction

*Staphylococcus aureus* is an important pathogen responsible for a broad range of human and animal diseases and toxin-mediated illnesses such as staphylococcal food poisoning and toxic shock syndrome (Tong et al., 2015; Zeaki et al., 2019). *S. aureus* pathogenic success is attributed to the possession of various virulence factors that include adhesins, immune evasion factors, enterotoxins, hemolysins, exfoliative toxins, Panton-Valentine leukocidin (PVL) and toxic shock syndrome toxin (TSST-1; Malachowa and DeLeo, 2010; Tong et al., 2015; Tam and Torres, 2019; Zeaki et al., 2019). In addition, *S. aureus* is able to acquire and develop resistance against various antimicrobial compounds including antibiotics frequently used in treatments such as beta-lactams. This is an attribute that limits therapeutic options and increases human and animal healthcare costs due to *S. aureus* infections (Malachowa and DeLeo, 2010; Nelson et al., 2018; Turner et al., 2019). Especially methicillin-resistant *S. aureus* (MRSA) strains are notorious and are routinely detected in healthcare (HA-MRSA), community (CA-MRSA) and livestock (LA-MRSA)-associated staphylococcal infections (Enright et al., 2002; Monecke et al., 2011; Lakhundi and Zhang, 2018).

In Africa, *S. aureus* infection is still considered to be one of the neglected diseases despite the acknowledged high burden of *S. aureus* infections within communities, healthcare settings and livestock production systems (Reddy et al., 2010; Herrmann et al., 2013; Schaumburg et al., 2014). Paucity of knowledge currently exists with respect to population structure, prevalence, characteristics and health burdens due to different *S. aureus* genotypes especially with respect to livestock and livestock-derived food products, particularly in Africa (Schaumburg et al., 2014; Lozano et al., 2016). Raw and spontaneously fermented milk products constitute an important nutrient source that is widely consumed by communities in Africa, particularly in rural and pastoral or agro-pastoral communities. These products also pose significant public health risks as potential vehicles for transmission of *S. aureus* and its toxins to human populations in general (Njage et al., 2013; Jans et al., 2017; Paudyal et al., 2017; Wambui et al., 2019; Owusu-Kwarteng et al., 2020). Also MRSA was reported in over 50% of strains isolated from bulk can milk and raw milk products and over 90% of strains carried at least one enterotoxin genes (Asiimwe et al., 2017) These public health risks are further exacerbated due to poor sanitation and milking hygiene practices, suboptimal milk handling and distribution infrastructures, as well as the existing high prevalence of human and intramammary dairy livestock colonization and infections with *S. aureus* (Owusu-Kwarteng et al., 2020; Seligsohn et al., 2020).

DNA sequence-based bacterial strain typing tools such as multilocus sequence typing (MLST) and whole genome sequence (WGS) are valuable tools in the interrogation of population structure, molecular epidemiology, dissemination routes and associated human and animal health risks of the different *S. aureus* genotypes (Enright et al., 2000; Harris et al., 2010). MLST assigns *S. aureus* strains to sequence types and clonal complexes (CC) based on the partial DNA sequence of the alleles of seven house-keeping gene loci. WGS in contrast uses the entire sequenced genome information for a more precise and high-resolution molecular typing of *S. aureus* strains (Enright et al., 2000; Harris et al., 2010). MLST-based interrogation of *S. aureus* population structures has previously revealed the clonal nature of *S. aureus* and identification of virulent MRSA and methicillin-sensitive *S. aureus* (MSSA) lineages as well as CC associated with the HA-MRSA, CA-MRSA and LA-MRSA strains (Enright et al., 2000, 2002; David and Daum, 2010; Tong et al., 2015).

We previously found that raw and fermented milk products derived from cows, goats and camels, products which are widely consumed by pastoral and sedentary communities in SSA, are also potential reservoirs for HA and LA *S. aureus* strains (Jans et al., 2017). DNA microarray-based analysis of such strains established that they were genetically diverse harboring various virulence and antibiotic resistance genes. Therefore, these strains may pose health risks for human consumption. We, however, currently lack high-resolution insights into the population structure and detailed genetic makeup of these strains. Generation of such information would be crucial in determining the public health risks posed by locally predominant *S. aureus* genotypes and their reservoirs. Furthermore, such information would contribute to the design of public health risk mitigation strategies in these widely consumed food products. In the present study, we aimed to utilize WGS to characterize this SSA milk-associated *S. aureus* strain collection and to provide information on their population structure and genetic composition and thus extrapolate on associated public health risks.

## Materials and methods

### Strain origin and growth conditions

African *S. aureus* strains (ILS03 to ILS79, *n* = 20, ILS = Institut für Lebensmittelsicherheit und –hygiene = Institute of Food Safety and Hygiene) were isolated from raw cow, camel and goat artisanal milk and their fermented products previously collected in Côte d’Ivoire, Kenya and Somalia between 2007 and 2014 (Jans et al., 2017; Table 1). Details on isolation source and milk sample types were previously published (Jans et al., 2012, 2013; Njage et al., 2013; Jans et al., 2017). Strains examined here were selected from the set of previously described strains based on representation of different product types and geographical origin. Reference strains for all laboratory work included *S. aureus* HG003, *S. aureus* Newman and *S. aureus* USA300. USA300 is a CA-MRSA (Diep et al., 2006) whereas HG003 is a NCTC8325 derivative with restored *rsbU* and *tcaR* genes (Herbert et al., 2010). Strains were routinely cultivated in Brain Heart Infusion (BHI) broth (Biolife, Milan, Italy) at 37°C under aerobic conditions. All materials were autoclaved at 121°C for 15 min.

**Table 1:** Genome statistics of *Staphylococcus aureus* strains of African dairy sources obtained and characterized in this study (genome sequences available under BioProject PRJNA310553 on GenBank).

Strain	Length (bp)	CDS	Genes	Source (contigs)	C + G (%)	Accession no.	Milk type	Milk origin	Country	Clade	Pres. association by clade (Monecke et al., 2011)	Orig. MLST association	MLST-ST	Nearest CC-ST	Yersiniabactin detected
ILS-03	2,753,650	2,735	2,798	61	32.7	LSFB00000000	Raw	Camel	Kenya	6	LA	Human	1738	CC707	Yes
ILS-06	2,789,685	2,792	2,851	59	32.7	LSFA00000000	Raw	Camel	Kenya	3*	CA & HA	Human	1741	CC5	Yes
ILS-09	2,743,719	2,731	2,795	61	32.7	LSFC00000000	Fermented	Camel	Somalia	5	CA & HA	Human	30	CC30	
ILS-12	2,788,672	2,831	2,880	59	32.7	LSAT00000000	Fermented	Camel	Somalia	6	LA	Livestock	1742	CC130	Yes
ILS-17	2,737,493	2,699	2,762	21	32.7	LSFD00000000	Raw	Camel	Kenya	6	LA	Livestock	1765	CC425	Yes
ILS-20	2,772,844	2,869	2,932	43	32.7	LSAS00000000	Raw	Camel	Kenya	3	CA & LA	Livestock	97	CC5-ST97	
ILS-26	2,746,298	2,789	2,852	74	32.7	LSAU00000000	Raw	Camel	Kenya	6	LA	Human	1781	CC707	Yes
ILS-37	2,807,976	2,895	2,960	72	32.7	LSAV00000000	Raw	Camel	Kenya	6	LA	Livestock	130	CC130	Yes
ILS-48	2,792,775	2,866	2,929	70	32.8	LSAW00000000	Fermented	Camel	Kenya	5	CA & HA	Human	30	CC30	
ILS-49	2,738,697	2,743	2,809	42	32.8	LSDV00000000	Fermented	Camel	Kenya	ST398	LA	Livestock	1952	CC398	
ILS-53	2,695,705	2,679	2,742	74	32.7	LSDW00000000	Raw	Camel	Kenya	3	CA & HA	Human	2013	CC5	
ILS-62	2,693,655	2,669	2,734	48	32.7	LSDX00000000	Fermented	Cow	Côte d’Ivoire	3	CA & LA	Human	72	CC5-ST72	
ILS-66	2,790,059	2,773	2,813	67	32.7	LSDY00000000	Raw	Camel	Kenya	5	CA & HA	Not done	30	CC30	
ILS-71	2,717,943	2,719	2,756	46	32.7	LSDZ00000000	Fermented	Cow	Côte d’Ivoire	3	CA & HA	Human	5	CC5	
ILS-72	2,715,617	2,762	2,815	81	32.6	NAZI00000000	Fermented	Cow	Côte d’Ivoire	3	CA & LA	Livestock	2077	CC5-ST9	
ILS-75	2,786,178	2,806	2,868	43	32.7	LSEA00000000	Fermented	Cow	Côte d’Ivoire	3	CA & HA	Human	5	CC5	
ILS-76	2,816,151	2,798	2,862	53	32.7	LSEB00000000	Raw	Goat	Kenya	5	CA & HA	Human	2080	CC30	
ILS-77	2,667,356	2,620	2,676	68	32.7	LSEC00000000	Fermented	Cow	Côte d’Ivoire	7	CA	Human	152	CC152	
ILS-78	2,704,187	2,685	2,750	70	32.6	LSED00000000	Fermented	Cow	Côte d’Ivoire	3	CA	Human	2094	CC88	
ILS-79	2,658,658	2,668	2,713	134	32.7	LSEE00000000	Fermented	Cow	Côte d’Ivoire	3	CA	Human	15	CC5-ST15	

CA, community associated; HA, hospital associated; LA, livestock associated; NV, Novel; 3*, separate branch not as part of clade 3, but due to visibility included in the figure of clade 3.

### DNA preparation

DNA for whole genome sequencing of the 20 *S. aureus* ILS strains was isolated from an overnight culture inoculated with material from a single colony. The extraction was performed as previously described using the DNAeasy Blood and Tissue Kit (Qiagen, Hilden, Germany; Jans et al., 2017).

### Genome sequencing of *Staphylococcus aureus* isolates

The genomes of all 20 *S. aureus* ILS isolates were sequenced at the Functional Genome Centre of ETH Zurich and the University of Zurich (Zurich, Switzerland). Libraries were prepared using the Nextera XT kit (Illumina Inc., San Diego, United States). MiSeq 2 × 150 bp platform and chemistry (Miseq V2 5–0-4) was used for genome sequencing (Illumina Inc.).

### Assembly and annotation of genomes

Genome assembly was performed using CLC Genomics Workbench *de novo* assembly algorithm (v11, Qiagen, Aarhus, Denmark) in multiple runs. Assemblies were performed after initial adapter trimming and read quality trimming using default settings. The option “map reads back to contigs” and combined with “update contig = yes” was activated for all assemblies. Assembly quality was checked in CLC and assembly stats.^1^ Annotation was performed using the NCBI Prokaryotic Genome Annotation Pipeline version 3.1. All annotated genomes were submitted to GenBank and are available under Bioproject ID PRJNA, BioSample ID range SAMN04452702-SAMN04452721 and corresponding GenBank accession No. as indicated in Table 1.

### Phylogenetic tree construction

Phylogenetic analysis was performed in several steps and using different analysis methods. Primarily, WGSA.net/pathogen.watch^2^ was used to obtain an overview of the phylogenetic relationships of the African *S. aureus* ILS strains in a global collection of *S. aureus* strains. To do so, 936 genomes out of >4,000 genomes in the Pathogen.watch database were selected based on representation of at least one member of each sequence type, each country and inclusion of all reference genomes. Individual strains per criteria were then selected at random whereas all strains identifiable as being of African origin were included. Together with the 20 ILS genomes of this study, this yielded a collection of 956 genomes (Supplementary Tables S1, S2) to calculate the core genome MLST (cgMLST) of *S. aureus*.

### Comparative analysis of genomes

Basic sequences statistics were performed using CLC Genomics Workbench (v11, Qiagen). MLST-ST confirmation was performed on MLST 2.0 (Larsen et al., 2012). Singleton calculations on strain and group levels were performed on EDGAR v2.3 using default settings and the automated homology cutoff calculations as described in the original publications (Blom et al., 2009, 2016). A reduced set of 79 strains was used for the initial comparison (Supplementary Tables S1, S3). The set was selected based on the two criteria “reference strain” and the origin of the strains being “Africa” to facilitate faster initial comparison calculations on presumably unique CDS. Those CDS identified as presumably unique based on this small strain set, were subsequently blasted against the available *S. aureus* genomes on NCBI Genbank to provide a comprehensive coverage.

Genomes were analyzed for virulence factors using all *S. aureus* genes archived within the virulence factor database VFDB (Chen et al., 2005). A bidirectional best hit approach was applied using blastp and an identity cutoff of 70%. Mobtyper (Robertson and Nash, 2018) and ResFinder (v4.1) were used to extract information on virulence factor genes (VFGs), plasmid replicons and antimicrobial resistance genes (ARGs) from the genome assemblies uploaded to the Center for Genomic Epidemiology^3^ (Zankari et al., 2012; Cosentino et al., 2013; Joensen et al., 2014). These online tools were applied using the default parameters for query coverage and percentage identity.

### Antibiotic sensitivity testing

The sensitivity of the 20 *S. aureus* ILS strains against a panel of six selected antibiotics was determined using the disk diffusion method. The assays were performed in duplicate on two separate occasions following the Clinical Laboratory Standards Institute (CLSI) guidelines (CLSI, 2017). The panel of tested antibiotics included bacitracin (0.04 units), ciprofloxacin (5 μg), cefoxitin (30 μg), penicillin (10 IU), teicoplanin (30 μg), fosfomycin (200 μg) and tetracycline (30 μg). The *S. aureus* USA 300 strain was used as a quality control to achieve the minimum standards dictated by the CLSI (2017).

### Screening for putative yersiniabactin siderophore operon carriers

Genomes of 928 *S. aureus* strains were screened for the presence of yersiniabactin operon structures. For this, the full operon DNA sequence was added to a blast-database in CLC Genomics workbench. This blast-database was then used to query the genomes of the 928 *S. aureus* strains using default settings. Hits of >1,000 bp and higher 90% sequence identity were visually confirmed for each strain prior to listing a strain as a carrier of the putative yersiniabactin siderophore operon.

For primer design and PCR development, genomes of strains harboring yersiniabactin-enconding genes were selected. The DNA sequence of the operon structure extracted from *S. aureus* strain ILS-03 in comparison with that of strain BSAR58 was used to identify conserved sequences. Four primer pairs were designed to target the four main genes comprised within the putative yersiniabactin siderophore-encoding operon (Table 2), namely the genes encoding for amp ligase (ILS-03 locus tag AXE88_09125), non-ribosomal peptide synthetase (AXE88_09130), polyketide synthase (AXE88_09135) and polyketide synthetase (AXE88_09145). Further genes upstream and downstream of the yersiniabactin siderophore-encoding operon were selected based on their conserved nature across yersiniabactin carrier and non-carrier strains. Genes encoding for the AraC family transcriptional regulator (AXE88_09195) and ATPase (AXE88_04705) were determined as conserved genes for this purpose and targeted using two additional primer pairs. All primers were designed in CLC Genomics Workbench with a universal nearest neighbor melting temperature of 58–60°C. All PCR assays were operated with 1 μM of each primer, 50 ng of DNA template and PCR master mix to a final volume of 25 μl. The PCR protocol encompassed an initial denaturation of 2 min at 95°C followed by 35 cycles of 30 s denaturation at 95°C, 30 s annealing at 56°C and 30 s replication at 72°C. PCR products were resolved on a 1.5% agarose gel and visualized under UV light after ethidium bromide staining.

**Table 2:** Primers for yersiniabactin locus detection in *S. aureus*.

Target gene (encoding protein)	Gene size (bp)	Locus	ILS03	Primer name	Primer sequence (5′–3′)	Expected amplicon size (bp)
Amp ligase	1′608	Yersiniabactin siderophore operon	AXE88_09125	Amp ligase fw	ATGCGATAGAAATGCAGAGAG	390
				Amp ligase rev	GTAAACCCATACTTTTATAACGTG	
Non-ribosomal peptide synthetase	6′018		AXE88_09130	lrp2-6 kb-fw	ATGTTACAAAAGGTGAAGTCTC	542
				lrp2-6 kb-rev	GTTTGTAAAATCGGCAACAATG	
Polyketide synthase	2′115		AXE88_09135	lrp3-fw	CCGAGCGATATTGATTTAGC	228
				lrp3-rev	TTCCATAAAATTTCGAACACTTG	
Polyketide synthetase	2′769		AXE88_09145	lrp2-2.7 kb-fw	AGGCATGAAGCTTTAAGGAC	309
				lrp2-2.7 kb-rev	TTCCACGCAATTATTATCGC	
AraC family transcriptional regulator	2′238	Downstream of yersiniabactin. Outside control	AXE88_09195	AraC-fw	GAGTTGTCTAAATTGACTGAGC	139
	2′238			AraC-rev	ACTCGATATAGTCGCATACTTG	
ATPase	3′153	Upstream yersinibactin. Outside control.	AXE88_04705	ATPase-fw	TATTCCGATGATTATGAGTGC	638
				ATPase-rev	TTTTGACAAATGCATCTTTAGG	

### Growth comparison under limited iron

Iron-limited growth conditions were generated as previously described (Ledala et al., 2014) by chelating divalent cations in TSB with 5% Chelex-100 (BioRad Laboratories, Hercules, California, United States) for 20 h at 4°C while stirring. Afterward, the Chelex was removed by filtrating through a 0.2-μm filter membrane (Thermo Fisher Scientific, Waltham, Massachusetts, United States). Defferated TSB was supplemented with 25 μM ZnSO_4_, 25 μM MnSO_4_, 1 mM MgSO_4_, 100 μM CaCl_2_, and 5 μM ferrous Iron (DTSB_fe_; Sigma-Aldrich Co., Missouri, United States). To compare growth, overnight cultures (16 h at 37°C and 150 rpm) of yersiniabactin siderophore operon-positive (*n* = 11) and negative (*n* = 18) *S. aureus* strains cultivated in normal TSB were harvested by centrifugation and washed in 10 ml PBS (Life Technologies Ltd., Paisely, United Kingdom) at 8,000 × *g* for 5 min. A 20-μl culture suspension in PBS standardized to an optical density 600 nm (OD_600_) of 1.0 for each strain was inoculated in 180 μl DTSB and TSB in 96-well microtiter plates (Corning Incorporated, New York, United States). Cultures were grown at 37°C with continuous medium shaking. Growth was monitored by measuring OD_600_ at 30-min intervals for 24 h in an Eon microtiter plate reader (BioTek, Lucerne, Switzerland). Each strain was assessed in three independent biological experiments that were performed in duplicates. Growth parameters, namely, lag phase (LPD), maximum growth rate (MGR), and area under the curve (AUC), in TSB and DTSB were determined from the OD-based growth curve data using the R package ‘opm’ as previously described (Wambui et al., 2020). Statistical analyses were performed using the program GraphPad Prism (version 9.3.1). DTSB growth parameters were all standardized to account for growth variability between strains in normal TSB without limited iron stress. To this end DTSB growth parameters of each strain were expressed relative to those determined in normal TSB. The Mann–Whitney U rank-sum test for nonparametric data was used to analyze statistical significant differences in standardized DTSB growth parameters (LPD, MGR, and AUC) between the groups of yersiniabactin operon positive and negative strains.

## Results

### Basic genome characteristics

The 20 genomes of *S. aureus* ILS strains obtained from camel, cow, and goat milk products in Côte d’Ivoire, Kenya, and Somalia featured a genome size of 2.66–2.82 Mb with a G + C content of 32.6%–32.7% and encoding for 2,676–2,960 genes (Table 1). Genome *de novo* assembly yielded 21–134 contigs (Table 1). Genome assembly statistics are provided in Supplementary Table S4.

### Phylogenetic analysis of strains and integration into a global perspective

#### cgMLST analysis of phylogenetic relationship among 956 *Staphylococcus aureus* isolates

**Figure 1:**
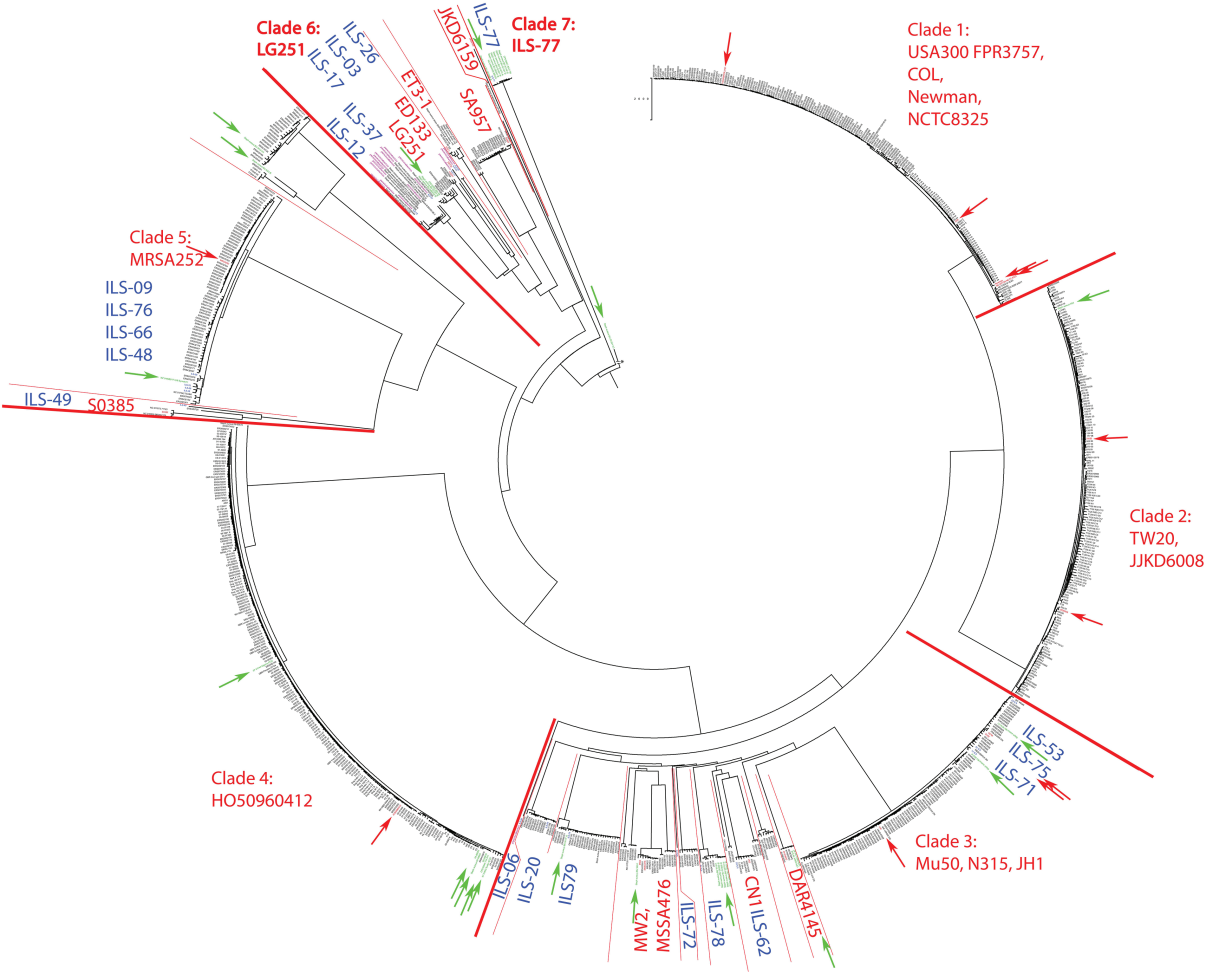
cgMLST tree of 956 *S. aureus* strains calculated on pathogen.watch using various reference *S. aureus* strains highlighted in each clade. For better visualization of the main phylogenetic tree structure, the branch length connecting the base of the tree (*S. aureus* BU G1201 t13 as the most distant strain) was cut from 854′621 units to 200 units and indicated accordingly. Blue indicates ILS strains reported in this study. Red boundaries and arrows indicate clade boundaries and tree location of all main reference strains, respectively. Green arrows indicate strains of African origin not originating from this study.

Analysis of the 20 ILS *S. aureus* strains and 936 *S. aureus* genomes selected from the pathogen.watch cgMLST identified seven clades of which five clades incorporated the majority of strains. These clades were represented by *S. aureus* reference strains USA300 FPR3757, COL, Newman and NCTC8325 (clade 1, mainly CC8, 186 isolates), TW20 and JKD6008 (clade 2, mainly CC239, 161 isolates), Mu50, N315, and JH1 (clade 3, mainly CC5, 213 isolates), HO50960412 (clade 4, mainly CC22, 208 isolates), and MRSA252 (clade 5, mainly CC30, 111 isolates; Figure 1). *S. aureus* strains derived from human sources in Africa (*n* = 33) were distributed among all clades except clade 1. The majority of the African human isolates clustered in clades 6 and 7 (*n* = 12), in or near clade 3 (*n* = 11), clade 4 (*n* = 6), clade 5 (*n* = 3), and clade 2 (*n* = 1).

Overall the 20 *S. aureus* ILS strains derived from African dairy products featured a selective distribution among several distinct clades whereas some strains formed novel clades. A large number of African isolates were directly part of clade 3 or clustered in close relationship to clade 3 together with human-derived African strains (Supplementary Figure S1). This included ILS-53, ILS-75, and ILS-71, which clustered within clade 3. ILS-06, ILS-20, ILS-79, ILS-72, ILS-78, and ILS-62 formed small diverging clades within clade 3 and in connection to reference strains MW2, MSSA476, CN1 and DAR4145. Members of clade 3 and adjacent clades were mainly assigned to CC5. Other ILS isolates (ILS-09, ILS-76, ILS-66, ILS-48, and ILS-49) clustered in close relationship to clade 5 and reference strain MRSA252 (Supplementary Figure S2). Clade 5 featured predominantly strains of CC30 (regularly associated with MRSA), but none of the ILS strains were found to be methicillin-resistant.

Furthermore, ILS-49, a member of the LA CC398, formed a separate clade adjacent to clade 5 defined by S0385. S0385 is a globally present LA-MRSA of sequence type (ST) 398 (Schijffelen et al., 2010; Supplementary Figure S2). ILS-49 was determined not to be methicillin-resistant.

**Figure 2:**
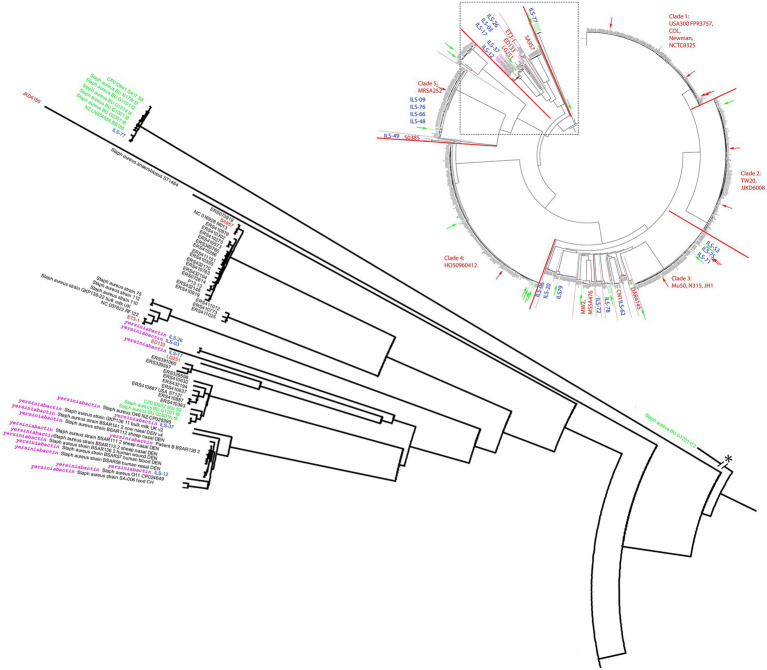
Detailed view of the *S. aureus* cgMLST tree clade comprised of strains carrying the putative yersiniabactin operon. The clade is extracted near the root of the cgMLST tree of 956 *S. aureus* strains (Figure 1). All putative yersiniabactin carriers are highlighted in purple. Strains derived from this study are highlighted with blue ILS-tags. Strains of African origin are highlighted in green. Reference strains are highlighted in red.

Near the base of the tree, the structure showed a diversification into multiple smaller clades summarized as clade 6 and comprised of reference strains LG251, ED133, ET3-1, SA957, and JKD6159 (Figure 2). ILS-03, ILS-26 (both CC707) clustered closest to ED133. ILS-17 (CC425) clustered closest to LG251 whereas ILS-37 and ILS-12 (both CC130) clustered in a separate clade comprised of European human, animal and dairy isolates. ILS-77 (CC152) together with eight African human isolates formed clade 7 comprised of only African-derived isolates. *S. aureus* BU G1201 t13 as the most distant isolate formed the base of the tree. For better visualization, its branch length was corrected from 854′621 units to 200 units. In this context, the branch length within isolates associated with LG251 as well as neighboring other clades suggest less homogeneity and more distant relationships among these isolates in contrast to the rather flat hierarchy observed in clades 1, 2, 4, and 5 as well as parts of clade 3. Clades neighboring LG251 seem to be in a region of the cgMLST tree that is still less represented by the selected sample collection and available genomes.

### Unique coding sequences among African *Staphylococcus aureus* dairy strains

#### CDS unique to the ILS strain pool

The 20 ILS strains represent a unique insight into dairy *S. aureus* of East and West Africa. In order to identify genes that might be shared among these ILS strains but that are unique among other *S. aureus* genomes, we performed a group-wise comparison. Using this approach, 225 genes (147 of which encoding for hypothetical proteins) were found to be unique in at least one ILS strain in comparison with the 79 other strains (Table 3). When expanding the grouping to merge ILS strains with all other African strains in comparison with strains outside of Africa, 431 CDS (227 encoding for hypothetical proteins) were found to be unique (Table 3). Subsequent NCBI Blast analysis of all genes presumptively identified to be unique among the limited strain pool of 20 ILS and 79 other *S. aureus* strains returned no major undescribed unique CDS. However, future research is needed to provide clarity into the multiple CDS still described as encoding for hypothetical proteins.

**Table 3:** Comparative genomics of 20 ILS *S. aureus* strains to 79 other *S. aureus* isolates selected according to their cgMLST relationship.

Strain name	Gene count	Yersiniabactin carrier	Overall unique genes	Unique genes among ILS strain pool by single ILS strains	Overall unique in Africa by single ILS	Overall unique in Africa by single ILS strains and found outside Africa	Overall unique genes of single ILS strains but shared with yersiniabactin-carrier strains	Overall unique genes of single ILS strains but shared with non-yersiniabactin-carrier strains outside Africa	Overall unique genes by ILS strain pool	Overall unique in Africa by ILS strain pool	Overall unique in Africa by ILS strain pool and found outside Africa	Overall unique genes of the ILS strain pool but shared with yersiniabactin-carrier strains	Overall unique genes of the ILS strain pool but shared with non-yersiniabactin-carrier strains outside Africa	Unique genes among ILS strain pool in comparison with outside Africa strain pool	Unique among ILSvs outside Africa reference strains but found among other African strains
ILS-03	2,798	yes	0 (0)	2 (1)	1 (1)	1 (1)	0 (0)	1 (1)	14 (7)	39 (20)	25 (13)	15 (7)	10 (6)	54 (29)	54 (29)
ILS-06	2,851	yes	8 (5)	20 (13)	17 (13)	9 (8)	3 (3)	7 (6)	21 (11)	51 (28)	30 (17)	20 (9)	13 (9)	24 (14)	16 (9)
ILS-09	2,795	no	0 (0)	7 (3)	2 (1)	2 (1)	1 (0)	2 (1)	0 (0)	3 (1)	3 (1)	1 (0)	3 (1)	11 (5)	11 (5)
ILS-12	2,880	yes	9 (9)	17 (15)	15 (15)	6 (6)	3 (3)	3 (3)	19 (13)	50 (32)	31 (19)	28 (16)	5 (4)	19 (13)	10 (4)
ILS-17	2,762	yes	2 (1)	4 (3)	3 (2)	1 (1)	0 (0)	1 (1)	5 (3)	31 (18)	26 (15)	21 (11)	6 (4)	7 (5)	5 (4)
ILS-20	2,932	no	4 (3)	23 (18)	7 (5)	3 (2)	0 (0)	3 (2)	7 (5)	11 (7)	4 (2)	0 (0)	4 (2)	25 (15)	21 (12)
ILS-26	2,852	yes	4 (3)	10 (7)	5 (3)	1 (0)	0 (0)	1 (0)	19 (11)	41 (20)	22 (9)	14 (6)	8 (3)	59 (34)	55 (31)
ILS-37	2,960	yes	3 (3)	10 (5)	4 (3)	1 (0)	0 (0)	1 (0)	14 (12)	40 (23)	26 (11)	24 (11)	6 (1)	22 (19)	19 (16)
ILS-48	2,929	no	5 (5)	9 (7)	5 (5)	0 (0)	0 (0)	0 (0)	15 (13)	23 (15)	8 (2)	1 (0)	8 (2)	25 (20)	20 (15)
ILS-49	2,809	no	12 (8)	32 (22)	29 (21)	17 (13)	6 (5)	13 (10)	15 (9)	42 (27)	27 (18)	11 (6)	19 (14)	32 (20)	20 (12)
ILS-53	2,742	no	3 (3)	9 (7)	4 (3)	1 (0)	0 (0)	1 (0)	3 (3)	4 (3)	1 (0)	0 (0)	1 (0)	3 (3)	0 (0)
ILS-62	2,734	no	2 (2)	4 (3)	3 (2)	1 (0)	0 (0)	1 (0)	4 (2)	5 (2)	1 (0)	0 (0)	1 (0)	6 (3)	4 (1)
ILS-66	2,813	no	2 (2)	5 (4)	2 (2)	0 (0)	0 (0)	0 (0)	17 (12)	27 (17)	10 (5)	3 (3)	10 (5)	22 (16)	20 (14)
ILS-71	2,756	no	0 (0)	5 (5)	0 (0)	0 (0)	0 (0)	0 (0)	0 (0)	1 (1)	1 (1)	0 (0)	1 (1)	2 (2)	2 (2)
ILS-72	2,815	no	38 (21)	47 (24)	38 (21)	0 (0)	0 (0)	0 (0)	39 (22)	45 (23)	6 (1)	1 (0)	6 (1)	44 (24)	6 (3)
ILS-75	2,868	no	4 (4)	12 (7)	6 (4)	2 (0)	0 (0)	2 (0)	4 (4)	9 (7)	5 (3)	0 (0)	5 (3)	9 (8)	5 (4)
ILS-76	2,862	no	0 (0)	2 (1)	0 (0)	0 (0)	0 (0)	0 (0)	17 (12)	28 (18)	11 (6)	3 (3)	11 (6)	24 (18)	24 (18)
ILS-77	2,676	no	0 (0)	30 (22)	3 (2)	3 (2)	2 (2)	1 (0)	2 (0)	5 (2)	3 (2)	2 (2)	1 (0)	16 (10)	16 (10)
ILS-78	2,750	no	8 (6)	23 (13)	11 (7)	3 (1)	2 (1)	3 (1)	10 (8)	25 (13)	15 (5)	5 (3)	15 (5)	20 (17)	12 (11)
ILS-79	2,713	no	0 (0)	11 (5)	1 (1)	1 (1)	0 (0)	1 (1)	0 (0)	2 (2)	2 (2)	0 (0)	2 (2)	7 (2)	7 (2)
Sum	56,297		104 (75)	282 (185)	156 (111)	52 (36)	17 (14)	41 (26)	225 (147)	482 (279)	257 (132)	149 (77)	135 (69)	431 (277)	327 (202)
Remaining non hypothetical genes			29	97	45	16	3	15	78	203	125	72	66	154	125

The first number indicates the number of genes in total followed by the number of hypothetical genes in brackets.

#### CDS unique among single ILS strains in comparison with other *Staphylococcus aureus* genomes

All 20 ILS isolates and 79 other *S. aureus* strains (Supplementary Table S1) selected from the different cgMLST clades were used for comparative genomics to perform a first screening for potentially unique CDS and patterns in African isolates. Among these 20 ILS and 79 other strains, ILS-06, ILS-49, ILS-72, and ILS-78 were the strains with the highest number of unique CDS overall. Only 104 CDS from these 20 ILS strains were fully unique, meaning that they appeared only once in the 20 ILS and 79 other *S. aureus* strains. Out of these 104 CDS, 75 CDS were identified as encoding for hypothetical proteins (Table 3). Only 29 CDS encoded proteins with annotated functions (Supplementary Table S3). These 29 CDS were further analyzed in comparison with GenBank, which found matching CDS at >95% sequence identity to CDS in other *S. aureus* strains for 12 CDS. Therefore, only a final set of 17 CDS originating from five ILS strains (ILS-06, ILS-17, ILS-49, ILS-72, and ILS-78) were found to be truly unique. Of these 17 CDS, 11 originated from ILS-72, seven of which were related to a putative *Staphylococcus*-plasmid encoding among others for type VI secretion proteins, plasmid functions and DNA binding. In ILS-06, the three unique CDS were all located adjacent to each other on the same contig. All three CDS were associated with transporter function. In ILS-17, one unique CDS was identified as encoding for a toxin-antitoxin system, whereas single unique CDS in ILS-49 and ILS-78 were identified as encoding for transcriptional regulators.

#### Unique CDS encoding for yersiniabactin, a putative siderophore

Interestingly, the in-depth analysis of seemingly unique CDS led to the discovery of a lineage of strains defined by the presence of a set of CDS termed to be involved in yersiniabactin synthesis. Yersiniabactin according to its annotated function is supposedly encoding for a siderophore involved in iron scavenging. The ILS strains identified as yersiniabactin carriers were: ILS-03, ILS-06, ILS-12, ILS-17, ILS-26, and ILS-37, all of which originated from Kenya or Somalia.

### Organization of the putative yersiniabactin-encoding genome region and its comparison among *Staphylococcus aureus* and closest relative

In order to investigate the presence of this putative siderophore, we analyzed 928 *S. aureus* genomes. Among the 928 strains queried in our cgMLST analysis, yersiniabactin CDS were limited to 18 strains, six of which being the ILS strains described above (Figure 2). The other strains identified as yersiniabactin carriers were BSAR57, BSAR58, BSAR111_2, BSAR112, BSAR113_2, BSAR136_2, BSAR141_2, GKP136-11, O11, O46, and SA-006 (Figure 2). Interestingly, the majority of strains carrying yersiniabactin were LA or food-associated strains (*n* = 14) with the exception of only three strains being associated with humans (BSAR57, BSAR58, and BSAR136_2). Furthermore, yersiniabactin seems to be present in two subclades within this main clade, but absent in two other subclades (Figure 2). Interestingly, ILS-06 seems to have a nearly identical yersiniabactin locus, but overall, the strain forms a very distinct separate new lineage adjacent to clade 3 and not clade 6. A reason for this distribution is so far not known.

**Figure 3:**
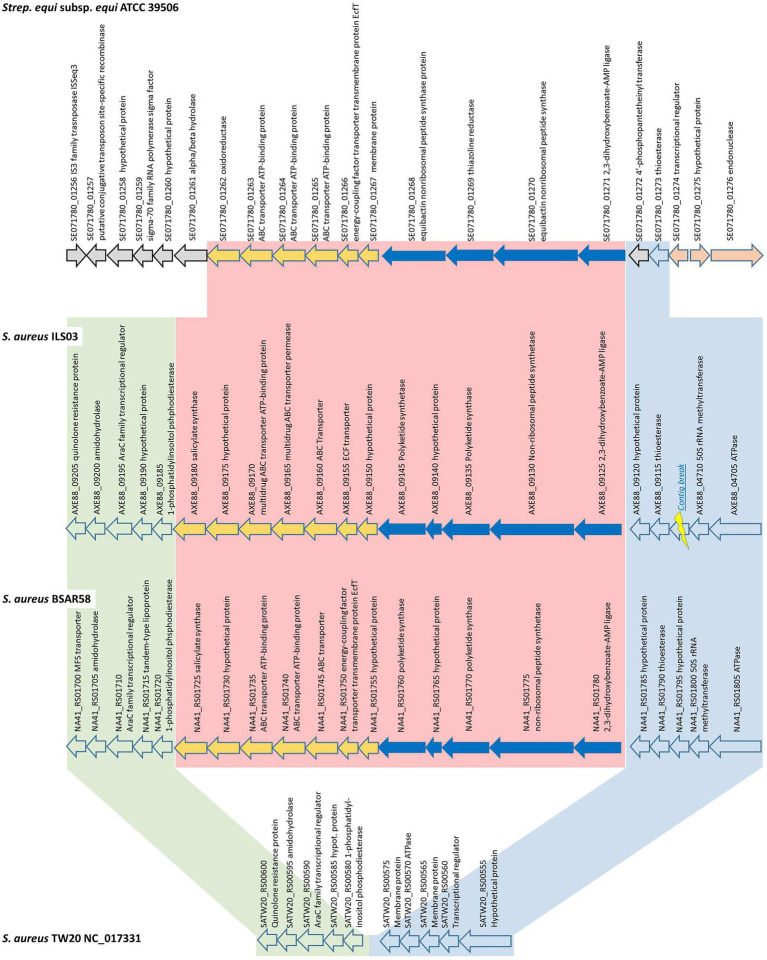
Detailed schematic overview of the putative yersiniabactin-encoding operon and short flanking regions shared among yersiniabactin-carriers (ILS-03 and BSAR58) and non-carrier (TW20) *S. aureus* strains as well as *S. equi* subsp. *equi* ATCC 39506 as closest other match.

A general comparison among several *S. aureus* strains (Figure 3) clustering in the same phylogenetic clade as ILS-03 revealed that this general organization of the yersiniabactin-encoding region is highly conserved among African (ILS-03, ILS-06, ILS-12, ILS-17, ILS-26, and ILS-37) and European *S. aureus* strains (BSAR57, BSAR58, BSAR111_2, BSAR112, BSAR113_2, BSAR136_2, BSAR141_2, GKP136-11, O11, O46, and SA-006).

In detail, the putative yersiniabactin-encoding genome region is comprised of 12 CDS from AXE88_09125 to AXE88_09180 using ILS-03 as a model strain (Figure 3). The first five CDS (AXE88_09125 to AXE88_09145) are the main CDS of the putative yersiniabactin production machinery and encode for putative AMP ligase, peptide synthetase, polyketide synthases, and hypothetical proteins. This is followed by seven CDS (AXE88_09150 to AXE88_09175) encoding mainly for different transporters, followed by AXE88_09180 encoding for a salicylate synthase.

To further analyze this genomic region, *S. aureus* TW20 (NC_017331) served as an interesting comparison in that it features only the conserved flanking regions but not the 12 yersiniabactin-related CDS (Figure 3; Supplementary Figure S3). A blast comparison of the genome section SATW20_RS00600-SATW20_RS00555 among multiple reference strains distributed among all major branches in our phylogenetic cgMLST analysis revealed that the genomic organization of TW20 is shared as well by strains US300_FPR3757, Mu50, JKD6159, CN1, MW2, MRSA252, and COL, featuring 94.6–99.9% sequence identity. This finding could in part support a hypothesis for this yersiniabactin region to be an insertion.

Following this idea of insertion, this prompted the search for other organisms sharing similar CDSs. A general blastp search on RAST suggested *Streptococcus equi* subsp. *equi* ATCC 39506 as a potential match with CDS identities and positive scores ranging between 26–56% and 49–77%, respectively (Supplementary Table S5). Subsequent bidirectional best hit comparison between ATCC 39506 and ILS-03 in CLC Genomics Workbench confirmed these matches for AXE88_01975 to AXE88_09125 including also two CDS (AXE88_09120 and AXE88_09115) directly adjacent in the upstream flanking region (Figure 3). AXE88_09115 encodes for a putative thioesterase linked possibly also to iron scavenging abilities. This shared region outside the actual region under investigation could contradict the above hypothesis of an insertion and would also support the hypothesis of a common ancestor with similar genetic organization and the subsequent deletion of the yersiniabactin-encoding region in all other strains.

### Screening of African and Swiss strain collection of livestock origin for yersiniabactin

**Figure 4:**
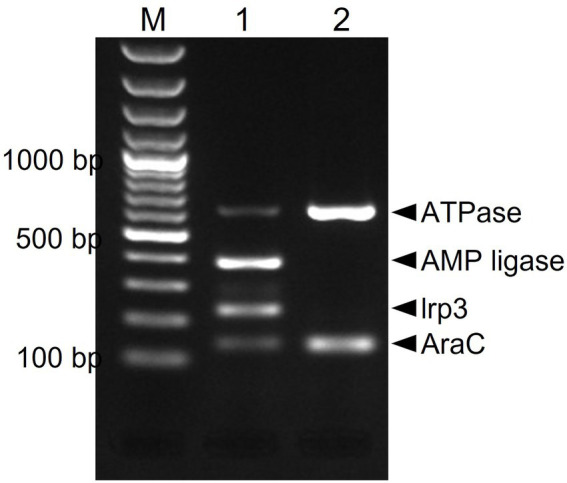
Multiplex PCR amplification and detection of the putative yersiniabactin operon genes in *S. aureus.* Primers targeting the AraC [139 bp], Lrp3 [228 bp], AMP ligase [390 bp], and ATPase [638 bp] genes were used. PCR amplicons were separated and visualized using agarose gel electrophoresis. M-100 bp DNA ladder, 1-ILS03 (positive control), and 2-ILS 09 (negative control).

Due to presumptive livestock-association of yersiniabactin carriage, we decided to screen our in-house African and Swiss *S. aureus* strain collections for the presence of several genes of the putative yersiniabactin operon. First, an *in silico* analysis was conducted revealing the presence of the yersiniabactin siderophore operon in six (ILS-03, ILS-06, ILS-12, ILS-17, ILS-26, and ILS-37) of the 20 African *S. aureus* genomes. To assess the distribution of this operon among the *S. aureus* isolates a multiplex PCR assay system targeting the genes encoding for ATPase, AMP ligase, lrp3, and AraC was developed and applied (Figure 4). This PCR assay correctly detected yersiniabactin operon presence in the six strains found to harbor this operon from *in silico* genome analysis (Supplementary Table S1). Its application to 51 *S. aureus* isolates from African fermented milk collection with yet un-sequenced genomes led to the detection of further 24 (24/51; 47%) more yersiniabactin siderophore operon carrying ILS strains (Supplementary Table S6). On the other hand, only one isolate (2.5%) tested positive for this operon when a collection of 43 Swiss mastitis milk *S. aureus* isolates available from the ILS strain collection was tested as an outgroup (data not shown).

### Elucidating the function of yersiniabactin as a putative siderophore

**Figure 5:**
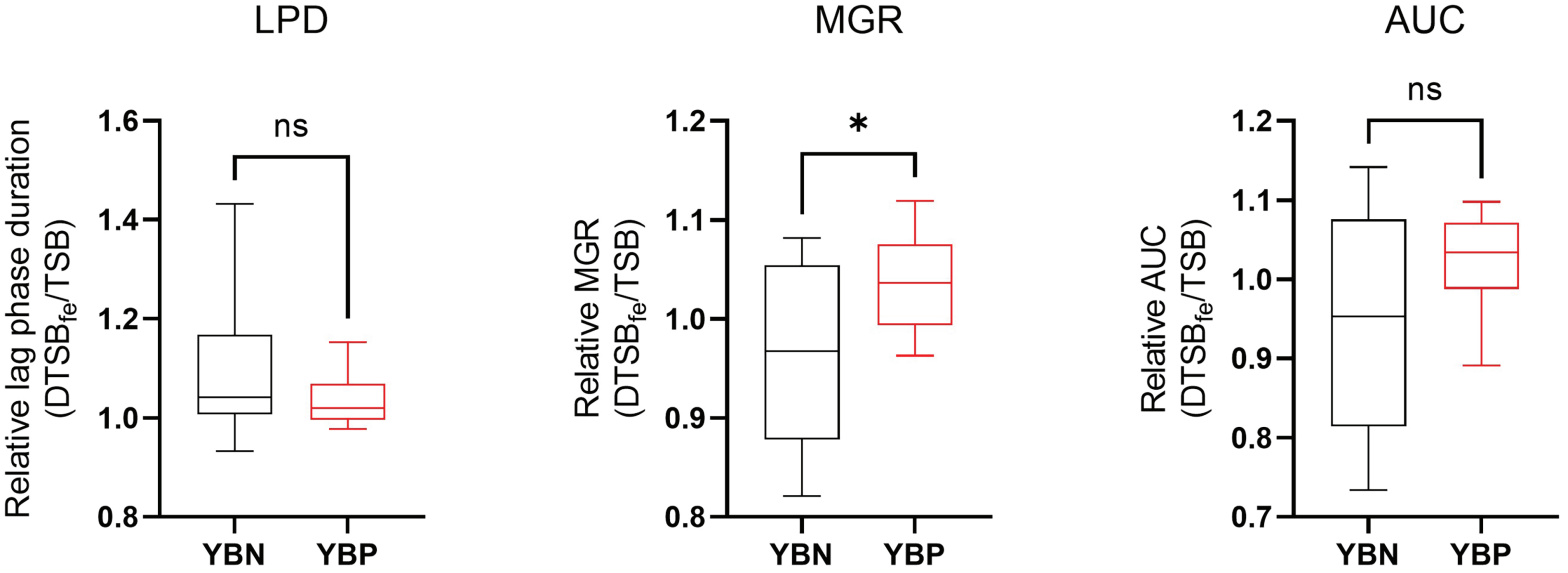
Yersiniabactin operon possession enhances growth rate under iron limited conditions in deferrated TSB media supplemented with 5 μM ferrous Iron (DTSBfe). Box plots comparison of relative growth parameters; area under growth curve (AUC), lag phase duration (LPD), and maximum growth rate (MGR) determined under limited iron conditions (5 μM Fe) in DTSB for the yersiniabactin negative (YBN; *n* = 18) and positive (YBP; *n* = 11) ILS *S. aureus* strain groups. Growth curve parameters determined under limited iron conditions (DTSBfe) were normalized to growth efficiency in normal TSB for each strain to correct for strain-dependent growth efficiency variation observed in this media. *Statistically significant differences (*P* < 0.05) between the YBP and YBN groups identified using the Mann–Whitney U test.

To assess the possible siderophore function for the discovered putative yersiniabactin operon we compared growth under limited iron availability conditions between a group of yersiniabactin operon carrying *S. aureus* strains (*n* = 11) and a control group comprising strains (*n* = 18) without this operon. Growth curve parameters total area under growth curves (AUC) capturing overall growth dynamics, lag phased duration (LPD), maximum growth rate (MGR), and final maximum cell density (MD), determined for each strain in deferrated TSB media (limited iron availability conditions), were normalized to correct for strain specific growth differences in non-deferrated control TSB broth. Comparing the normalized parameters between the two strain groups revealed that although the yersiniabactin operon positive strains as a group displayed slightly higher AUC and shorter LPD medians than the yersiniabactin negative control strain group, such differences were not statistically significant (Figure 5). On the other hand comparing MGR revealed that the yersiniabactin operon strain group had significantly higher (*p* < 0.05; Mann–Whitney U test) MGRs than the control strain group without this operon during growth under limited iron availability conditions (Figure 5) This observation thus suggests that yersiniabactin operon carrying strains possess a growth fitness advantage than strains without the operon under the limited iron availability conditions applied in our studies. The discovered putative yersiniabactin operon might thus be beneficial for growth of *S. aureus* strains possessing this operon under limited iron availability.

### Virulence factors, antimicrobial resistance and risk prediction

VirulenceFinder analysis of the 20 African *S. aureus* genomes revealed that ILS strains harbored between 5 and 21 of the known virulence factor genes (VFGs; Table 4; Supplementary Tables S6, S7). Besides a core set of 5 VFGs possessed by all the 20 strains, there were 16 VFGs, which were variably distributed among the strains including enterotoxin, toxic shock syndrome, leukotoxin, and epidermal cell differentiation inhibitor genes. Panton-Valentine leucocidin genes (*lukS/F-PV*) were present in three (ILS-09, ILS-77, and ILS-79) strains, whereas the leukotoxin DE genes (*lukD/E*) were harbored in 12 strains. One (ILS-53) and four (ILS-12, ILS-37, ILS-72, and ILS-77) strains, respectively, carried the toxic shock syndrome (*tst1*) and epidermal cell differentiation inhibitor B (*edinB*) genes. The staphylococcal complement inhibitor (*scn*) gene was detected in seven (ILS-09, ILS-20, ILS-53, ILS-62, ILS-71, ILS-75, and ILS-79) strains, whereas eight strains, most (7/8) of which had the *egc* enterotoxin gene cluster (*seg*, *sei*, *sem*, *sen*, *seo*, and *seu*) carried enterotoxin genes. The enterotoxin A (*sea*) gene was detected in five (ILS-48, ILS-53, ILS-66, ILS-75, and IL-S79) strains. SED (*sed*), SEJ (*sej*) and SER (*ser*) genes were detected in a single strain ILS-75, which also harbored *sea* and the *egc* cluster genes. PlasmidFinder detected seven plasmid replicon types among the 20 African *S. aureus* strain genomes with 15 (75%) genomes containing between one and three plasmid replicon types, whereas five genomes had none (Table 4; Supplementary Table S6). ARG screening using ResFinder revealed that 9 of the 20 genomes contained between one and eight resistance genes targeting aminoglycosides (*aad* and *aa6*; *n* = 3), tetracyclines (*tetK*; *n* = 8), beta-lactams (*blaZ*; *n* = 5), quinolones (*grlA* and *gyrA*; *n* = 1), and sulphonamides (*dfrG*; *n* = 1). Meanwhile examining the sequence environment associated with the tetracycline (*tetK*) and beta-lactam resistance (*blaZ*) ARGs showed that they were located on four plasmid types based on contig size and sequence composition. Most of the *tetK* genes were located on a 4-kb contig harboring plasmid replicon type 7a but in one strain (ILS-75) the gene was located on large 36-kb contig harboring a rep 7a type plasmid replicon, β-lactam, bleomycin and cadmium resistance genes as well as *sed* and *sej* enterotoxin genes. The β-lactam resistance genes in three strains ILS-62, ILS-20, and ILS-09 was located on contigs carrying plasmid replicon types rep7c, 7a, and 20 origins, respectively, as well as cadmium resistance and bacteriocin transporter genes. One strain (ILS-79) had β-lactam resistance genes located together with bacteriocin transporter genes on 20-kb contig carrying a plasmid replicon type rep 7a origin. Phenotypic resistance testing against selected antibiotics showed that all strains were sensitive to fosfomycin and teicoplanin but resistant to bacitracin (Supplementary Table S6). There were eight, five and one strain, respectively, that were resistant to tetracycline, penicillin and ciprofloxacin, which harbored corresponding ARGs in agreement with the genomic resistance profiles inferred from ResFinder genome analysis (Table 4).

**Table 4:** ILS *S. aureus* strains and their genotype and phenotype details on plasmids, antimicrobial resistance, and virulence factors.

Strain	Country of origin	Plasmid genotype	Antimicrobial resistance genotype	Antimicrobial resistance phenotype	Virulence factor genotypes (VFGs)
ILS-03	Kenya	rep16, rep19			*aur*, *hlgC*, *hlgB*, *hlb*, *hlgA*
ILS-06	Kenya	rep16, rep19			*aur*, *splE*, *hlb*, *hlgA*, *hlgC*, *hlgB*, *lukE*, *lukD*
ILS-09	Somalia	rep16, rep5a	*blaZ*	PEN	*aur*, *splE*, *sak*, *scn*, *seo*, *sem*, *sei*, *seu*, *sen*, *seg*, *hlb*, *hlgA*, *hlgC*, *hlgB*, *lukF-PV*, *lukS-PV*
ILS-12	Somalia				*aur*, *splA*, *splB*, *sak*, *edinB*, *hlgA*, *hlgC*, *hlgB*, *hlb*, *lukD*, *lukE*
ILS-17	Kenya				*aur*, *splA,splB*, *splE*, *hlgA*, *hglC*, *hlgB*, *hlb*, *lukD*, *lukE*
ILS-20	Kenya	rep7a, rep20	*blaZ*, *tetK,str*	PEN, TET, STR	*aur*, *splA*, *splB*, *splE*, *sak*, *scn*, *hlgB*, *hlgC*, *hlgA*, *hlb*, *lukD*, *lukE*
ILS-26	Kenya				*aur*, *hlgA*, *hlgB*, *hlgC*, *hlb*
ILS-37	Kenya	rep16, rep19, repUS13			*aur*, *splA*, *splB*, *splE*, *sak*, *hlgA*, *hlgC*, *hlgB*, *hlb*, *edinB*, *lukD*, *lukE*
ILS-48	Kenya	rep16, rep19			*aur*, *sak*, *sea/sep*, *hlb*, *hlgA*, *hlgB*, *hlgC*
ILS-49	Kenya				*aur*, *hlb*, *hlgA*, *hglB*, *hlgC*
ILS-53	Kenya	rep7a, repUS21	*tetK*	TET	*aur*, *splA*, *splB*, *scn*, *sak*, *sea/sep*, *sei*, *sem*, *sen*, *seo*, *seu*, *hlb*, *hlgA*, *hlgB*, *hlgC*, *lukD*, *lukE*, *tst*
ILS-62	Côte d’Ivoire	rep7c, rep20	*blaZ*, *tetK*	PEN, TET	*aur*, *splA*, *splB*, *splE*, *scn*, *sak*, *sei*, *seg*, *sem*, *sen*, *seo*, *seu*, *hlb*, *hlgA*, *hlgB*, *hlgC*, *lukD*, *lukE*
ILS-66	Kenya	rep16, rep19			*aur*, *splE*, *sak*, *sea/sep*, *seg*, *sei*, *sem*, *sen*, *seo*, *seu*, *hlb*, *hlgA*, *hlgB*, *hlgC*
ILS-71	Côte d’Ivoire	rep7a	*tetK*	TET	*aur*, *splA*, *splB*, *scn*, *sak*, *seg*, *sei*, *sem*, *sen*, *seo*, *seu*, *hlb*, *hlgA*, *hlgB*, *hlgC*, *lukD*, *lukE*
ILS-72	Côte d’Ivoire	rep7a	*tetK*, *str*	TET, STR	*aur*, *splA*, *splB*, *splE*, *hlb*, *hlgA*, *hlgB*, *hlgE*, *lukD*, *lukE*, *edinB*
ILS-75	Côte d’Ivoire	rep7a, rep20, rep22	*aac6*, *aaD*, *blaZ*, *dfrG*, *tetK*, *blaZ*, *glrA p.S80F*, *gyrA p.S84L*	PEN, TET, CIP, GENT	*aur*, *hlb*, *hlgA*, *hlgB*, *hlgC*, *sak*, *splA*, *splB*, *scn*, *sea/sep*, *sed*, *seg*, *sei*, *sej*, *sem*, *sen*, *seo*, *ser*, *seu*, *lukD*, *lukE*
ILS-76	Kenya	rep16, rep19, rep7	*tetK*	TET	*aur*, *hlb*, *hlgA*, *hlgB*, *hlgC*, *splE*, *seg*, *sei*, *sem*, *sen*, *seo*, *seu*
ILS-77	Côte d’Ivoire				*aur*, *hlb*, *hlgA*, *hlgB*, *lukS-PV*, *lukF-PV*, *edinB*
ILS-78	Côte d’Ivoire	rep16			*aur*, *hlb*, *hlgA*, *hlgB*, *hlgC*, *splA*, *splB*, *lukD*, *lukE*
ILS-79	Côte d’Ivoire	rep5a. rep7a, rep16	*blaZ*, *dfrG*, *tetK*	PEN, TET	*aur*, *hlb*, *hlgA*, *hlgB*, *hlgC*, *sea/sep*, *scn*, *splA*, *splB*, *splE*, *lukD*, *lukE*, *lukF-PV*

## Discussion

Our study aimed at presenting the first insight into genetic makeup of African food-borne *S. aureus* strains and their population structure through WGS and comparative genomics. Initial characterization on these strains was previously performed through classic MLST and single gene analysis tools (Jans et al., 2017). With the growing wide availability of WGS, we wanted to use this technology for a much more in-depth look at the phylogeny and genetic makeup of these food-borne isolates in comparison with other HA and LA strains of African and global origin. This is also due to the importance of *S. aureus* as a human and animal pathogen as well as a causative agent for a neglected tropical disease (Herrmann et al., 2013).

In this context, it was important to elucidate the phylogenetic relationship of food-borne *S. aureus* and whether food-borne isolates are more likely to be related to CA, HA, or LA *S. aureus* lineages in the African setting. Given the current selection of isolates, phylogenetic analysis allowed for no clear trend to be identified toward any of these three reservoirs as nearly equal numbers of isolates relating to putative CA, HA, and LA lineages were observed. This observation based on WGS data confirms our previous observations on these strains using *spa*-typing and MLST (Jans et al., 2017).

Importantly, this observation supports previous hypotheses on the role of raw milk in the distribution of *S. aureus* between human and animal sources as well as within the human or animal reservoir or ecosystem (Tomao et al., 2020; van den Brom et al., 2020). Interestingly, these reports mainly observed the issue of LA lineages being present in milk (Tomao et al., 2020) or the limited development of animal-derived *S. aureus* infections in humans (Boss et al., 2016). However, the presence not only of LA strains including ST-398 but also CA and HA strains in East and West African milk products suggests a concern for animal and human health alike. Furthermore, it supports the need to classify *S. aureus* infection as important but so far a neglected tropical disease (Herrmann et al., 2013). Good manufacturing practices during milk production, processing as well as animal welfare would therefore be of great importance for animal and human health alike (van den Brom et al., 2020).

Our population structure analysis confirms and expands previous findings on *S. aureus* population structure observed in Africa (Ruffing et al., 2017). Our study also indicates that the majority of African isolates are assigned to CCs 5, 15, and 30. These CCs relate to typical *S. aureus* isolates of Europe and Africa (Ruffing et al., 2017). Interestingly, however, despite that the largest subgroup of ILS isolates belong to CC5 (8 out of 20 isolates), which represents also the predominant CC in the African healthcare setting and a main reservoir for MRSA (Abdulgader et al., 2015), none of the ILS isolates were MRSA. This suggest that at least in the environment and food setting in the countries studied in this work and in contrast to Uganda (Asiimwe et al., 2017), MRSA might not be as prevalent as elsewhere. On the other hand, ILS-77 and ILS-78 were classified as members of CCs 152 and 88, respectively, two CCs reported to be typical for African *S. aureus* (Abdulgader et al., 2015; Ruffing et al., 2017; Kyany'a et al., 2019). In case of CC152, eight other African isolates formed a distinct African clade with ILS-77, all of which belong to ST152. CC152 is significantly associated with HA and in part also CA strains in Africa (Ruffing et al., 2017) supporting again the potential role of milk as reservoir for various *S. aureus* lineages. In addition, isolates belonging to previously undescribed CCs 130, 398 and 425 for the African ecosystem were isolated from milk. Particularly the detection of a ST-398 strain, which is related to a highly transmissible clone (Uhlemann et al., 2012), might be concerning regarding its distribution in the community *via* milk. On a positive note, at least within our strain set, no USA300/ST8 isolates were found, although they are known to have established in the communities in SSA (Strauss et al., 2017). Although our isolate set was small and probably not representative of the entire SSA *S. aureus* population, these findings showed some clear distinctions from the otherwise expected typical strain portfolio of African community-sourced isolates. Our findings suggest that the population structure of *S. aureus* in SSA is still in many ways unknown and likely diverse across SSA countries as observed for example in milk products from some regions of Uganda, where a much higher MRSA-prevalence was detected (Asiimwe et al., 2017). Our findings of milk as a reservoir for CA, HA and LA strains furthermore highlight the need for an integrative approach such as that under the One Health concept in order to reduce the burden of *S. aureus* on animals and humans.

Interestingly, the overall genetic makeup of the 20 ILS *S. aureus* strains was not significantly different from the publicly available other *S. aureus* genomes globally. This was reflected in the small number of CDS identified as unique to these strains most of which do not yet possess an annotated function. Therefore, the ILS strains still contributed to the open pan genome of *S. aureus* (Bosi et al., 2016) even though the rate of added new genes per strains seems to be minor.

Despite the small number of unidentified CDS, by utilizing clade-specific markers, we observed differences that were shared mainly within a clade but so far not further described. One such feature was the detection of a putative siderophore operon annotated as yersiniabactin and the identification of a main “Yersiniabactin clade.” This clade is mainly comprised of LA-isolates of African and a few European sources. The isolates were identified as members of CCs 707, 130, 425 with the exception of ILS-06 being assigned to CC5. Siderophores have been described in *S. aureus* in the past such as staphyloferrin A and B (Conroy et al., 2019) which was identified, e.g., in ILS-03. However, yersiniabactin was previously not described in *S. aureus* and no other blast hits could be found. The closest link was established by sequence identity and genetic organization to *S. equi*. It remains, however, unclear, whether this was an insertion or deletion event. Given the complete absence of any remaining pseudogenes or genetic leftovers in other *S. aureus* strains, we hypothesize this yersiniabactin to be an insertion, for which not sufficient numbers of isolates are yet available to reconstruct the evolutionary tree. Our newly developed PCR-test identified at least one other LA-isolate of Swiss origin and could be helpful in finding answers. In support of its annotated role in iron acquisition, we showed by comparing strain growth dynamics *in vitro* that strains possessing this operon might have a growth fitness advantage compared to those without as they displayed a higher growth rate under limited iron availability conditions. We thus hypothesize that possession of this operon might provide growth advantage to *S. aureus* strains within infected hosts when iron availability is limited. The hypothesized role of this operon in iron acquisition would, however, need to be examined through further experimental work including confirmation experiments conducted using knock-out (isogenic deletion) and complemented mutants of *S. aureus* strains. The hypothesis of a growth advantage under iron limited conditions was, however, previously already suggested in connection with *Bifidobacterium* strains isolated from iron deficient Kenyan infants (Vazquez-Gutierrez et al., 2015).

In addition to providing a high-resolution genetic population structure of these SSA milk-associated *S. aureus* strains, WGS further confirmed previously described public health risks of such strains as many of them possess virulence factors and ARGs. Although the strain set analyzed was comprised entirely of MSSA strains, the examined strains also harbored resistance genes against other antibiotics commonly used in human and veterinary medicine such as penicillin and tetracycline. Although some differences in distribution and genetic sequences of some virulence factors and ARGs were observed, there were no obvious genetic features identified that are unique to the examined SSA *S. aureus* strain collection. Typical virulence factors identified included *scn*, a marker gene encoding for an immune evasion cluster (IEC) frequently associated with human origin *S. aureus*, as well as genes encoding for classical food poisoning-associated enterotoxins and the toxic shock syndrome (TSST-1). Moreover, three strains harbored *pvl* showing that significant populations of methicillin-sensitive, *pvl*-positive *S. aureus* strains that were reported for African healthcare settings may have extended their reservoir beyond the clinical setting (Abdulgader et al., 2015). In combination with the genotypically identified antimicrobial resistance, which in many cases were phenotypically confirmed, these findings support our previous conclusions and further stresses the need to further investigate non-clinical *S. aureus* in order to tackle *S. aureus* infections as a neglected tropical disease (Herrmann et al., 2013). It is, however, also important to point out that our evaluation of virulence and antibiotic resistance genes within the examined set of SSA *S. aureus* strain genomes is by no means exhaustive. Our genetic comparison is limited to the extent of the current information that is available within the publicly available databases that were used in our study. It is thus also expected that, besides variation in the genetic elements identified here, the SSA *S. aureus* genomes examined also possess further genetic variation in other putative virulence and ARGs that are not captured within the databases queried in our study.

In conclusion, the population structure, genotypic makeup, presence of virulence factors and antimicrobial resistance supports the theory of milk as a reservoir for *S. aureus* in SSA as many of the features reported to be typical for African clinical *S. aureus* isolates could be observed in milk isolates. Given the role of *S. aureus* infections as neglected tropical disease, the impact of *S. aureus* on human and animal health alike and as such also on the livestock economy, an integrative One Health approach is required for future success. Comparative genomics in this case again helped to identify novel traits such as the putative yersiniabactin siderophore and to develope detection methods. Despite the vast number of available isolates and whole genome sequences, detailed characterization on genome sequence level still proves to be highly beneficial and supportive toward a better understanding of *S. aureus*, even if such isolates were previously already analyzed using MLST or other typing schemes.

## Data availability statement

The datasets presented in this study can be found in online repositories. The names of the repository/repositories and accession number(s) can be found in the article/Supplementary material.

## Author contributions

CJ and TT wrote the grant application and the manuscript draft, designed the experiments, supervised lab work, and performed analyses and interpretation of data for comparative genomics, phylogeny, genetic composition and link to phenotype data. JW performed and interpreted phenotypic and genotypic antimicrobial resistance and siderophore experiments. MS performed the analysis on plasmids, ARGs, and virulence genes and provided subsequent interpretations. All authors contributed to manuscript revision, read, and approved the submitted version.

## Funding

This study was funded by the World Food System Center of ETH Zurich, Switzerland and the Institute of Food Safety and Hygiene from the University of Zurich. This study was based on the reanalysis of milk samples previously collected during the frame of projects funded by the North–South Centre of ETH Zurich and the UBS Optimus Foundation in Côte d’Ivoire, Kenya and Somalia.

## Conflict of interest

The authors declare that the research was conducted in the absence of any commercial or financial relationships that could be construed as a potential conflict of interest.

## Publisher’s note

All claims expressed in this article are solely those of the authors and do not necessarily represent those of their affiliated organizations, or those of the publisher, the editors and the reviewers. Any product that may be evaluated in this article, or claim that may be made by its manufacturer, is not guaranteed or endorsed by the publisher.

## Footnotes

^1^https://github.com/sanger-pathogens/assembly-stats (Accessed July 20, 2022).

^2^https://pathogen.watch/ (Accessed July 20, 2022).

^3^http://www.genomicepidemiology.org/ (Accessed July 20, 2022).
